# Cardiometabolic multimorbidity and symptoms of common mental disorders in a primary healthcare system for low-income urban population in Pakistan

**DOI:** 10.1371/journal.pgph.0005140

**Published:** 2025-09-09

**Authors:** Rafay Iqbal, Asra Qureshi, Rabia Jaffar, Wajiha Omair, Asif Ali Imam, Unab Inayat Khan

**Affiliations:** 1 Department of Family Medicine, Aga Khan University, Karachi, Pakistan; 2 Department of Family Medicine, Indus Hospital & Health Network, Karachi, Pakistan; 3 SINA Health, Education & Welfare Trust, Karachi, Pakistan; University of Alberta Faculty of Medicine & Dentistry, CANADA

## Abstract

The objective of the study was to evaluate the prevalence of cardiometabolic multimorbidity and symptoms of common mental disorders, including depression, anxiety, and stress, and association between the two, in primary care clinics for low-income, urban population in Karachi, Pakistan. We performed a descriptive cross-sectional study at two primary healthcare clinics, catering predominantly to low-income families in Karachi. Adults, aged 30 years and above, coming to the two clinics for acute illnesses or for regular follow-up of chronic conditions were recruited. We used convenience sampling to include participants who consented. We excluded pregnant women and individuals presenting with severe acute symptoms. Of the 496 participants, 406 (82%) were women. Cardiometabolic multimorbidity was present in 231 (47%) participants, with diabetes in 297 (60%) and hypertension in 259 (52%) being the most common cardiometabolic conditions. Anxiety, stress, and depression affected 462 (93%), 387 (78%), and 335 (67%) participants, respectively, being higher in women. Only a small percentage of individuals were referred to mental health services (13% at site-1 and 16% at site-2). The study did not find a significant positive association between cardiometabolic multimorbidity and symptoms of common mental disorders. This study highlights the high prevalence of cardiometabolic multimorbidity and symptoms of common mental disorders in a low-income urban population visiting primary care clinics in Pakistan. To better understand the true prevalence of cardiometabolic multimorbidity and its association with mental disorders, future community-based prospective studies with representative population are recommended.

## Introduction

Cardiometabolic diseases (CMD) are prevalent worldwide, with diabetes mellitus affecting an estimated 11.1% of adults, hypertension 33%, hypercholesterolaemia 39% and cardiovascular diseases around 8%, [1–4]. Cardiometabolic multimorbidity (CMM), is defined as co-existence of two or more of these CMDs in an individual [5]. The prevalence of CMM has increased significantly over time; in the United States (US) adults, it has risen from 9.4% in 1999–2000 to 14.4% in 2017–2018, whereas in China, it increased from 2.41% to 5.94% over five years till 2016 [6,7]. People with CMDs often present with multiple conditions and according to a South Asian study, almost 25% of patients with hypertension were affected by CMM [8]. Literature has shown that CMM is associated with an increased risk of cognitive dysfunction [9], all-cause mortality [10], reduced quality of life, and higher healthcare costs [11].

Common mental disorders, including anxiety and depression, are highly prevalent globally affecting 29.2% individuals over a lifetime [12]. Global Burden of Disease (GBD) data reported that 3.9% of the world’s population experienced mental disorders in 2021. Mental disorders constitute 17% of the total years lived with disability (YLDs) globally; with depressive disorders and anxiety being the second and sixth highest causes of YLDs respectively [13]. According to GBD report, 71% of global burden of anxiety disorder could be avoided through access to treatment. Anxiety disorders have a substantial impact on cognitive functioning and social relationships [14]. Functional impairment is found in 60% of individuals suffering from depression, affecting daily life activities, interpersonal relationships and workplace performance [15,16]. Common mental disorders are rapidly rising globally and are found to be more prevalent in low- and middle-income countries (LMICs), defined by the World Bank as the countries with Gross National Income (GNI) per capita of up to $14,005 [17,18]. In Pakistan, the prevalence of depression and anxiety was found to be 33.6% in community-based studies [19]. In an elderly tribal population of India, prevalence of anxiety was reported at 8.2% and depression at 22.4% [20]. Although the prevalence of mental disorders is high, access to mental healthcare is poor. As Pakistan has a limited number of trained mental health professionals (0.14 psychiatrists per 100,000 population), care for common mental disorders such as depression, is provided mainly by primary care physicians [21,22].

There is increased risk of depression, anxiety and stress in people with individual CMDs. For example, in people with cardiac disease, prevalence of depression has been reported as 31.3%, anxiety 32.9% and stress 57.7% [23–25]. Furthermore, people with CMM have a higher risk of depression, with cumulative effect of number of CMDs [26–28]. However, there is limited data examining this association among the Pakistani population. A study from the same clinical set up has looked at prevalence of mental disorder in patients with multimorbidity, with conditions including hypothyroidism, joint pain and skin infections, but among CMD, only hypertension and diabetes were included [29]. A similar community-based study from Karachi, Pakistan, defined multimorbidity more broadly, not only including CMDs but a wide range of other conditions like anaemia, thyroid disease, dyspepsia etc [30]. A study from 47 low- and middle-income countries, investigated association between multimorbidity and anxiety & depression, including conditions like asthma, back pain, hearing loss etc.; however, it only included angina and diabetes among CMDs [31].

Prevalence of individual CMD in Pakistan is well reported, with diabetes affecting 31.4% of adults, hypertension 44%, dyslipidaemia 73.9% and CVD 18.9% [1,32–34]. However, there is limited data on prevalence of CMM, especially in an urban population. Therefore, the objective of this study was to identify the prevalence of CMM and symptoms of common mental disorders, along with their socio-demographic correlates in patients visiting a primary healthcare system catering to a low-income urban population of Karachi, a major city of Pakistan. A secondary objective was to assess the association between CMM and symptoms of common mental disorders.

Based on the data cited above and given that this is a clinic-based sample, we hypothesized that the prevalence of both CMM & symptoms of common mental disorders will be high in this population with a positive association between the two.

## Methods

### Ethics statement

The study was conducted after approval by the ethical review board of the education & welfare trust (approval no ERB0000017/09–23). Written informed consent was obtained from all study participants.

### Study design & settings

This descriptive cross-sectional study was conducted at two primary care clinics in Karachi, operated by a local education and welfare trust. It is a not-for-profit organization that provides healthcare to less privileged communities through primary care clinics in Karachi. It has established 38 clinics in impoverished areas of the city, offering subsidized primary care services to low-income communities. The clinics are run by general practitioners. We selected the clinics based on patient volume, accessibility and relevance to the study objective. Both clinics have an average daily patient flow of 100–125 with almost 40–50% of them having CMDs. To compare distribution of symptoms of depression, anxiety and stress, we selected one clinic that offers counselling service, located in the North Nazimabad area of the city (site-1), and the other without (in Baldia area;site-2).

### Study population

Study population comprised of adults, aged 30 years and above, coming to the two clinics for acute illnesses (e.g., acute infections, musculoskeletal problems etc.) or for regular follow-up of chronic conditions including diabetes, hypertension, stroke, ischemic heart disease and dyslipidaemia. The age range of 30 years and above was selected due to the low prevalence of CMM in individuals below this age [30,35].

### Recruitment & eligibility criteria

We used convenience sampling to recruit participants from both sites. All adults (over 30 years of age), who attended the clinics during the recruitment period were considered eligible. Those who agreed to participate were included in the study and a written informed consent was obtained. We excluded pregnant women as the unique physiological changes of pregnancy may affect the outcome measures and may not be comparable to the population under study. We also excluded individuals presenting with severe acute symptoms as their immediate medical needs and potential underlying diagnoses differ from chronic conditions under investigation.

### Study measures

#### Cardiometabolic multimorbidity.

Cardiometabolic multimorbidity was defined as the co-existence of two or more of the following CMDs: hypertension, diabetes, dyslipidemia, ischemic heart disease, and stroke [5]. Participants self-reported history of CMDs when asked if they had ever been diagnosed with any of these conditions, and year of diagnosis was recorded. This was later verified from medical records for a documented diagnosis, use of medications, blood pressure checks and investigations.

#### Symptoms of common mental disorder (depression, anxiety, and stress).

Depression, anxiety, and stress were measured with the Depression, Anxiety, and Stress Scale-21 (DASS-21) [36,37]. This is a 21-item screening tool that gives a separate score for the three emotional states. Items are scored on a four-point Likert scale to assess symptoms experienced over the past week. Scores are categorized according to five severity ranges: ‘normal’, ‘mild’, ‘moderate’, ‘severe’, and ‘extremely severe’. DASS-21 has been used widely in studies across diverse populations and has been shown to translate well across cultures. We used the Urdu version for this study, which is the national language of Pakistan. This has been validated in different areas of Pakistan with similar cultural and economic background, showing a reliable Cronbach’s alpha of 0.93 [38,39].

### Sample size calculation

The sample size computation was based on the prevalence of depression among patients with CMM as 32% and 20% for patients without CMM [40]; using a two-sided confidence level of 95%, a power of 80% (1-β) and a 1:1 ratio of patients with and without CMM, the minimum effective number was 452; which was inflated by 10% to account for missing data. We recruited 501 participants, with almost half from each site. OpenEpi v3.01 online calculator was used for computations [41].

### Data collection

A structured questionnaire was designed electronically on REDCap® to collect data. One of our two data collectors had a bachelor’s degree with data collection experience in community-based surveys, including mental health assessment. The other was a final year medical student. Both were proficient in local languages. They were trained by the research team on all aspects of data collection, including gathering information about CMD, administration of DASS-21 and extraction of data from medical records. After obtaining informed consent, participants were interviewed to capture details including a) demographics b) clinical details (risk factors for disease, CMDs, and clinical parameters) and c) depression, anxiety, and stress scores. Medical records were checked, to verify diagnosis and medications and get investigation results.

### Statistical analysis

Data were reviewed for completeness and consistency before statistical analysis. We used descriptive statistics to summarize the socio-demographic characteristics of participants, chronic conditions, and screening for common mental disorders, using frequencies and percentages for categorical variables, mean ± standard deviation (SD) for normally distributed variables, and median (with interquartile range) for skewed continuous variables.

A count variable for CMDs, ranging from 0 (no disease) to 5 (maximum) was created to assess participants for CMM. Scores on DASS-21 were multiplied by 2 and the recommended conventional severity levels (i.e., normal, mild, moderate, severe, and extremely severe) were followed [42].

We performed descriptive analysis to examine site-specific differences in socio-demographic characteristics of study participants. Furthermore, we compared risk factors, CMDs, CMM, and symptoms of common mental disorders assessed through DASS-21 across the two study sites. Cross-tabulations using the *χ*^*2*^ test of association and *t-test* for two independent samples were performed to examine possible associations and comparison of mean scores for symptoms of common mental disorders. All analyses were performed using Stata/SE 18 (StataCorp, College Station, TX, USA), with statistical significance set at a two-tailed *p-value of less than* 0.05.

### Patient and public involvement statement

No patients or members of public were involved in the design or conduct of this study.

## Results

Data were collected between November 2023 and March 2024. A total of 569 patients were approached, of which 501 consented (response rate 88%) and analysis was performed on 496 participants with complete information (five participants were excluded as they met the exclusion criteria). Table 1 shows the site-specific socio-demographic characteristics of study participants. Majority of the participants were females (406, 82%) across the two study sites. The mean age of participants was 49.2 ± 12 years with no significant difference across the two clinic sites (*p = 0.145*). Women were younger (47.5 ± 11.5 years), as compared to men (57 ± 10.7 years). Family size was smaller for participants from site-2, 5.5 ± 2.5 as compared to site-1 participants, 6.7 ± 3.5 (*p* < 0.01). Almost half (n = 253) owned the property they lived in, and the rest rented. Educational attainment was very low, with 328 of the participants (66%; 32% men, 73% women) reported not receiving any formal education. Only 54 of the participants (11%) were employed; 94% of women were homemakers and 57% of men were unemployed. In this low-income study population, 319 participants (63.6%) had a household income of rupees 25,000 or less. Based on this, all were eligible for subsidized care. Only 190 participants (38%; 80% men, 29% women) owned cell phone, 28 (15%) of whom had a smartphone. Only 36 participants (7%) reported internet access.

**Table 1:** Site-specific socio-demographic characteristics of study participants.

Characteristics	Total N = 496	Site-1 n = 249 (50.2%)	Site-2 n = 247 (49.8%)	*p-*value
**Sex**				
Female n (%)	406 (82)	193 (77)	213 (86)	0.012^*^
**Age (years)** mean ± SD	49.2 ± 12	50.0 ± 11.4	48.4 ± 12.4	0.145^**^
**Marital status **n (%)				
Single	3 (1)	2 (1)	1 (0.4)	0.373^*^
Married	390 (78)	202 (81)	188 (76)	
Others (divorced, widowed, refused)	103 (21)	45 (18)	58 (24)	
**Spoken language** n (%)				
Urdu	78 (16)	52 (21)	26 (11)	<0.01^*^
Pashto	301 (61)	180 (72)	121 (49)	
Punjabi	37 (7)	7 (3)	30 (12)	
Hindko	61 (12)	3 (1.2)	58 (23)	
Others^†^	19 (4)	7 (3)	12 (5)	
**Family size** (mean ± SD)	6.1 ± 3.1	6.7 ± 3.5	5.5 ± 2.5	<0.01^**^
**Housing** n (%)				
Owned	253 (51)	121 (48)	132 (53)	0.28^*^
Rental	243 (48)	128 (51)	115 (46)	
**Education** n (%)				
No education	328 (66)	168 (67)	160 (64)	<0.07^*^
Informal education	9 (2)	2 (1)	7 (3)	
Primary (grade 1–5)	71 (14)	31 (12)	40 (16)	
Middle (grade 6–8)	38 (8)	26 (10)	12 (5)	
Secondary (grade 9–10)	29 (6)	12 (5)	17 (7)	
College and above	21 (4)	10 (4)	11 (4)	
**Employment** n (%)				
Employed^††^	54 (11)	30 (12)	24 (9)	0.17^*^
Homemaker	382 (77)	185 (74)	197 (79)	
Retired	6 (1.2)	2 (0.8)	4 (2)	
Unemployed	54 (11)	32 (13)	22 (9)	
**Monthly household income (PKR)** n (%)				
No income	57 (11)	22 (9)	35 (14)	<0.01^*^
≤ 10,000	3 (0.6)	2 (1)	1 (0.4)	
>10 000 - ≤ 25000	259 (52)	132 (53)	127 (51)	
> 25000	66 (13)	52 (19)	14 (5.6)	
Preferred not to say	111 (22)	41 (16)	70 (28)	
**Cell phone ownership** n (%)				
Yes	190 (38)	88 (35)	102 (41)	0.173 ^*^
Smartphone users	28 (15)	17 (19)	11 (11)	0.098^*^
**Internet access** n (%)	36 (7)	24 (10)	12 (5)	0.040^*^

^†^Sindhi, Siraiki, Balochi, Bengali, Kohistani.

^††^government employee, private job, self-employed/ business.

*χ2 square test of independence **Two independent sample t-test.

Table 2 compares the site-specific risk factors, CMDs, CMM, and symptoms of common mental disorders. The mean body mass index (BMI) of the study population was 28.1 kg/m^2^, with participants from site-1 having a higher BMI value (28.8 ± 6.3 kg/m^2^) than those at site-2 (27.5 ± 5.7 kg/m^2^) (*p* = 0.015). Tobacco use (cigarettes smoking and chewing tobacco) was reported only by 52 participants (10%). Overall, 231 participants (47%) had CMM, with a significant site-specific difference (*p* < 0.01), being higher at site-1 (n:152; 61%) compared to site-2 (79; 32%). The most prevalent CMD was diabetes mellitus in 297 participants (60%), followed by hypertension in 259 (52%) and dyslipidaemia in 101 (20%), all three being significantly higher (*p* < 0.01) at site-1*.* Using DASS-21, the most prevalent symptoms was anxiety in 462 participants (93%), followed by stress in 387 (78%) and depression in 335 (67%). Furthermore, the symptoms were significantly higher among participants from site-2 compared to site-1, with anxiety in 235 (94%) versus (vs) 227 (91%), stress in 216 (87%) vs 171 (68%) and depression in 207 (84%) vs 128 (49%). Importantly, there were significantly higher number of individuals in extremely severe categories for each of these mental illnesses, particularly at site-2. However, only 13% of individuals at site-1 and 16% at site-2 reported being referred to mental health clinics.

**Table 2:** Site-specific distribution of risk factors, CMDs, CMM, and symptoms of common mental disorders.

Characteristics	Total N = 496	Site-1 n = 249 (50%)	Site-2 n = 247 (49.8%)	*p-*value
**BMI (kg/m**^**2**^) mean ±SD	28.1 ± 6.0	28.8 ± 6.3	27.5 ± 5.7	0.015^**^
**Tobacco use n(%)**				
Cigarette smoking	11 (2)	5 (2)	6 (2.4)	0.75^*^
Chewing tobacco (niswar, gutka)	41 (8)	22 (9)	19 (7)	0.64^*^
**Systolic blood pressure mmHg** mean ± SD *(n:494)*	122.7 ± 16.1	127.1 ± 16.7	118.3 ± 14.1	<0.01^**^
**Diastolic blood pressure mmHg** mean ± SD *(n:494)*	79.3 ± 9.6	82.4 ± 9.7	76.1 ± 8.4	<0.01^**^
**Hypertension**	259 (52)	164 (66)	95 (38)	<0.01^*^
**Diabetes Mellitus**	297 (60)	170 (68)	127 (51)	<0.01^*^
**Dyslipidaemia**	101 (20)	76 (30)	25 (10)	<0.01^*^
**Ischaemic heart disease**	20 (4)	13 (5)	7 (3)	0.17^*^
**Stroke**	21 (4)	13 (5)	8 (3)	0.27^*^
**Number of CMDs**				
0-disease	133 (27)	42 (17)	91 (37)	<0.01^*^
1-disease	132 (26)	55 (22)	77 (31)	
2-diseases	138 (28)	85 (34)	53 (21)	
3-diseases	83 (17)	58 (23)	25 (10)	
≥4-diseases	10 (2)	9 (4)	1 (0.4)	
**CMM** (≥2 CMDs)	231 (47)	152 (61)	79 (32)	<0.01^*^
**DASS-21 Depression**				
Mild	49 (10)	34 (13)	15 (6)	<0.01^*^
Moderate	79 (16)	39 (15)	40 (16)	
Severe	35 (7)	16 (6)	19 (8)	
Extremely severe	172 (34)	39 (15)	133 (54)	
**DASS-21 Anxiety**				
Mild	32 (6.4)	22 (9)	10 (4)	<0.01^*^
Moderate	24 (5)	19 (8)	5 (2)	
Severe	35 (7)	24 (10)	11 (4)	
Extremely severe	371 (75)	162 (65)	209 (84)	
**DASS-21 Stress**				
Mild	38 (8)	27 (11)	11 (4)	<0.001^*^
Moderate	87 (17)	56 (22)	31 (12)	
Severe	94 (19)	48 (19)	46 (19)	
Extremely severe	168 (34)	40 (16)	128 (52)	

*χ2 square test *of* independence; **Two independent sample t-test.

Table 3 presents sex-specific differences in CMD, CMM, and symptoms of common mental disorders. Although not statistically significant (*p = 0.098*), CMM was higher in males (54%) compared to females (45%). Among CMDs, diabetes was significantly higher in males compared to females (75% vs. 56%; *p* = 0.001). Furthermore, the likelihood of screening positive for depression, anxiety, and stress was significantly higher (*p < 0.01*) in females compared to males. Prevalence of hypertension, diabetes and CMM was significantly higher with increasing age, but age didn’t significant affect the prevalence of symptoms of depression, anxiety and stress.

**Table 3:** Sex-specific distribution of CMD, CMM and symptoms of common mental disorders.

Disease	Total N=496	Male n = 90 (18%)		Female n = 406 (82%)		*p-value* ^*^
	n (%)	n (%)	95% CI	n (%)	95% CI	
**Hypertension**	259 (52)	54 (60)	49.1 to 70.1	205 (50)	45.5 to 55.4	0.10
**Diabetes mellitus**	297 (60)	68 (75)	65.3 to 84.4	229 (56)	51.4 to 61.2	0.001
**Dyslipidaemia**	101 (20)	19 (21)	13.2 to 30.9	82 (20)	16.3 to 24.4	0.84
**Stroke**	21 (4)	4 (4)	1.2 to 10.9	17 (4)	2.4 to 6.6	0.91
**Heart Attack**	20 (4)	4 (4)	1.2 to 10.9	16 (4)	2.2 to 6.3	0.82
**CMM** ^**+**^	231 (46)	49 (54)	43.6 to 64.9	182 (45)	39.9 to 49.8	0.098
**DASS-21 – Depression**	335 (67)	48 (53)	42.5 to 63.9	287 (71)	66–75	0.001
**DASS-21 – Anxiety**	462 (93)	76 (84)	75.2 to 75.2	386 (95)	92.4 to 96.9	<0.001
**DASS-21 – Stress**	387 (78)	56 (62)	51.3 to 72.2	331 (81)	77.4 to 85.1	<0.001

*χ2 test *of* independence; CI: confidence interval.

Table 4 examines association between CMM and symptoms of common mental disorders and provides sex-specific comparison of DASS-21 mean scores. We did not find any significant associations except for depression (*p* = 0.021), which in fact, showed a negative relation. We also examined association of CMM with mean DASS-21 scores, stratified by sex. The overall mean scores of all three common mental disorders were higher in females compared to males. Again, there were no significant associations except for depression in females, and that too showing a negative relationship.

**Table 4:** Association between CMM and symptoms of common mental disorders and sex-specific comparison of DASS-21 mean scores.

DASS-21	Without CMM n = 265 (53%)	With CMM n = 231 (47%)	*p-value* ^*^	DASS-21	Without CMM n = 265 (53%)	With CMM n = 231 (47%)	*p-value* ^**^
	**n (%)**	**n (%)**			**mean ± SD**	**mean ± SD**	
Depression	191 (72)	144 (62)	0.021	**Male**	10 ± 9.0	7.1 ± 6.7	0.09
				**Female**	11.0 ± 7.4	9.4 ± 7.2	0.025
Anxiety	245 (92)	217 (94)	0.51	**Male**	12.8 ± 9.2	12.3 ± 8.2	0.76
				**Female**	16.4 ± 8.2	15.9 ± 7.7	0.46
Stress	214 (81)	173 (75)	0.11	**Male**	13.2 ± 10.2	10.3 ± 7.0	0.11
				**Female**	14.8 ± 7.3	13.7 ± 7.2	0.10

*chi-square test of independence; ** t-test for two independent samples.

## Discussion

This study aimed to evaluate the prevalence of CMM and symptoms of common mental disorders along with their association in two clinics providing primary health care in low-income urban communities. It provides useful insights into the healthcare needs of this study population that already faces multiple barriers due to low literacy and poverty. Presence of subsidised care through the primary care clinics of the welfare trust, fulfils the basic health needs of many such households. All the participants would be eligible to avail subsidized government health services, but they regularly attend these clinics, both for acute minor illnesses and chronic disease management. This highlights the gap in the healthcare system.

There was a high percentage of CMM and symptoms of common mental disorders. Cardiometabolic multimorbidity was reported by 47% of the study participants and a further 26% had one CMD. Our study population had a high BMI, a recognized risk factor for CMM [43]. Tobacco use, an associated risk factor [5], was reported by only 10% of study participants, in contrasts to tobacco use prevalence in urban areas of Pakistan of 16% [44].

This may have either been because of lower use due to financial constraints, underreporting or generally lower use among women.

There was a high prevalence of symptoms of common mental disorders with anxiety being the most prevalent (93%), followed by stress (78%) and depression (67%). With such high frequencies of diseases that impact all-cause mortality, quality of life and healthcare expenditure, the results are an alarming portrayal of the healthcare needs of these low-income urban communities [45,46].

Our sample consisted of a significantly higher number of females (82%). This is likely because the clinics operate in the morning to mid-afternoon, when women are more likely to access them, while men who are employed are likely to be at work. This may explain why 57% of men who presented to the clinics were unemployed. Women were disadvantaged by lower education, employment status and mobile phone ownership. Given the socio-economic status of our study population, education level was much lower compared to national literacy rate of 49% [47].

Low literacy and socioeconomic status may explain why only 15% participants had a smartphone and even fewer had internet access, the so-called digital divide, which has important implications for health care interventions in this population [48]. Yet, these results are not at par with other studies conducted in rural areas of Northern Pakistan, where 80% of women reported having a mobile phone [49]. The lower mobile phone ownership in this study may have been affected by underreporting due to cultural factors like family’s disapproval for females to access modern technology [50].

In our study 47% of participants had CMM, which is higher compared to CMM prevalence of 10.2% in individuals with hypertension reported from the rural areas of Pakistan [8]. The same study reported CMM prevalence of 36.3% in Sri Lanka and 27.4% in Bangladesh. A prevalence of 5.94% have been reported from China and 14% from United States in 2018 [6,7]. The reason for a higher percentage in our population is that this data is from clinics providing management of CMD. As expected, CMM was more common in older individuals in our study. Although not statistically significant, more males had CMM. This is likely because males were older in our study.

Our data showed a high percentage of anxiety (93%), stress (78%) and depression (67%), with a significant proportion in very severe categories, which is much higher than prevalence of these conditions worldwide and in Pakistan [30,51]. This reflects the impact of socio-economic factors on mental health of this population [52]. This also shows high prevalence of unrecognized symptoms of common mental disorders, as majority of these individuals presented with non-mental health related illnesses. Consistent with global trends, higher percentage of women had anxiety, stress and depression in our study sample [53]. We selected two sites to see if presentation of symptoms of common mental disorders differs between one with counselling services (site-1) and the other without. Extremely severe symptom category of all three mental disorders was higher at site-2. This is likely because site-2 had higher percentage of females who had higher overall percentage of symptoms of common mental disorders. Referral to mental health clinic was low (13% at site-1 and 16% at site-2), despite availability of the facility at site-1. This reflects that most of these symptoms were unrecognized.

Contrary to previous studies, [26–28] this study did not find a significant positive association between CMM and common mental disorders. There could be a number of factors for this. Symptoms of common mental disorders, especially anxiety, were very high in this sample, likely driven by factors other than CMM, like adverse socio-economic status. Secondly, our study sample comprised mostly of women, who were relatively younger and had higher prevalence of symptoms of depression, anxiety and stress and lower prevalence of CMM compared to men.

## Limitations

A cross-sectional study design was used as a pragmatic approach to get a snapshot of the current situation. However, to establish causality, a longitudinal study design will be more appropriate. Our data was collected from primary care clinics, introducing a bias towards individuals seeking healthcare. We used convenience sampling as a feasible strategy under resource constraints of a real-world primary care set up, which may limit external validity of our study. Randomization or stratification can make the results more generalizable. Our population comprised of more females than males, which can skew results due to a difference in protective factors, risk factors and socio-demographic variables. These two primary care clinics operate during the morning to mid-afternoon, hence working individuals, especially employed men, can be assumed to have attended less. Despite these limitations, our findings highlight the high prevalence of CMM, anxiety, stress and depressionin this urban clinic-based population, with potential for extrapolation in prospective community-based studies in future.

## Conclusion

This study showed a very high prevalence of both CMM and symptoms of common mental disorders, especially anxiety. Furthermore, all symptoms of common mental disorders were more prevalent in women whereas CMM was not significantly different between sexes. This suggests factors beyond CMM that are contributing to women’s mental health.

This study adds to the limited literature available on CMM prevalence in Pakistan. This has implications for the health system planning as CMM is associated both with increased healthcare utilization and mortality. The study has further identified high prevalence of unrecognized symptoms of common mental disorders, in a low-income urban population. This highlights the need to screen for these illnesses for early diagnosis and intervention. To identify true prevalence of CMM and its association with mental disorders, community-based prospective studies including a representative population are needed.

## Supporting information

S1 Data Anonymized participant-level data used in the analyses of cardiometabolic multimorbidity and mental health among a low-income population. (XLSX)

## References

[1] International Diabetes Federation. Diabetes Atlas, 11th edn. Brussels, Belgium: IDF; 2025 [cited 2025 April 24]. Available from: https://diabetesatlas.org

[2] World Health Organization. Global Report on Hypertension: WHO; 2023 [cited 2025 April 24]. Available from: https://www.who.int/publications/i/item/9789240081062

[3] World Heart Federation. Cholesterol [Available from: https://world-heart-federation.org/what-we-do/cholesterol/

[4] British Hearth Foundation. Heart Statistics 2024 [cited 2025 10 Mar]. Available from: https://www.bhf.org.uk/what-we-do/our-research/heart-statistics

[5] Otieno P, Asiki G, Wekesah F, Wilunda C, Sanya RE, Wami W, et al. Multimorbidity of cardiometabolic diseases: a cross-sectional study of patterns, clusters and associated risk factors in sub-Saharan Africa. BMJ Open. 2023;13(2):e064275. doi: 10.1136/bmjopen-2022-064275 36759029 PMC9923299

[6] Zhang D, Tang X, Shen P, Si Y, Liu X, Xu Z, et al. Multimorbidity of cardiometabolic diseases: prevalence and risk for mortality from one million Chinese adults in a longitudinal cohort study. BMJ Open. 2019;9(3):e024476. doi: 10.1136/bmjopen-2018-024476 30833320 PMC6443196

[7] Cheng X, Ma T, Ouyang F, Zhang G, Bai Y. Trends in the Prevalence of Cardiometabolic Multimorbidity in the United States, 1999-2018. Int J Environ Res Public Health. 2022;19(8):4726. doi: 10.3390/ijerph19084726 35457593 PMC9027860

[8] Feng L, Jehan I, de Silva HA, Naheed A, Farazdaq H, Hirani S, et al. Prevalence and correlates of cardiometabolic multimorbidity among hypertensive individuals: a cross-sectional study in rural South Asia-Bangladesh, Pakistan and Sri Lanka. BMJ Open. 2019;9(9):e030584. doi: 10.1136/bmjopen-2019-030584 31488490 PMC6731877

[9] Lyall DM, Celis-Morales CA, Anderson J, Gill JMR, Mackay DF, McIntosh AM, et al. Associations between single and multiple cardiometabolic diseases and cognitive abilities in 474 129 UK Biobank participants. Eur Heart J. 2017;38(8):577–83. doi: 10.1093/eurheartj/ehw528 28363219 PMC5381595

[10] Emerging Risk Factors Collaboration, Di Angelantonio E, Kaptoge S, Wormser D, Willeit P, Butterworth AS, et al. Association of Cardiometabolic Multimorbidity With Mortality. JAMA. 2015;314(1):52–60. doi: 10.1001/jama.2015.7008 26151266 PMC4664176

[11] Han Y, Hu Y, Yu C, Guo Y, Pei P, Yang L, et al. Lifestyle, cardiometabolic disease, and multimorbidity in a prospective Chinese study. Eur Heart J. 2021;42(34):3374–84. doi: 10.1093/eurheartj/ehab413 34333624 PMC8423468

[12] Steel Z, Marnane C, Iranpour C, Chey T, Jackson JW, Patel V, et al. The global prevalence of common mental disorders: a systematic review and meta-analysis 1980-2013. Int J Epidemiol. 2014;43(2):476–93. doi: 10.1093/ije/dyu038 24648481 PMC3997379

[13] GBD 2021 Diseases and Injuries Collaborators. Global incidence, prevalence, years lived with disability (YLDs), disability-adjusted life-years (DALYs), and healthy life expectancy (HALE) for 371 diseases and injuries in 204 countries and territories and 811 subnational locations, 1990-2021: a systematic analysis for the Global Burden of Disease Study 2021. Lancet. 2024;403(10440):2133–61. doi: 10.1016/S0140-6736(24)00757-8 38642570 PMC11122111

[14] Craske MG, Stein MB, Eley TC, Milad MR, Holmes A, Rapee RM, et al. Anxiety disorders. Nat Rev Dis Primers. 2017;3:17024. doi: 10.1038/nrdp.2017.24 28470168 PMC11009418

[15] McKnight PE, Kashdan TB. The importance of functional impairment to mental health outcomes: a case for reassessing our goals in depression treatment research. Clin Psychol Rev. 2009;29(3):243–59. doi: 10.1016/j.cpr.2009.01.005 19269076 PMC2814224

[16] Hammer-Helmich L, Haro JM, Jönsson B, Tanguy Melac A, Di Nicola S, Chollet J, et al. Functional impairment in patients with major depressive disorder: the 2-year PERFORM study. Neuropsychiatr Dis Treat. 2018;14:239–49. doi: 10.2147/NDT.S146098 29386897 PMC5767094

[17] Friedrich MJ. Depression Is the Leading Cause of Disability Around the World. JAMA. 2017;317(15):1517. doi: 10.1001/jama.2017.3826 28418490

[18] The World Bank. World Bank Country and Lending Groups 2025 [cited 2025 April 27]. Available from: https://datahelpdesk.worldbank.org/knowledgebase/articles/906519-world-bank-country-and-lending-groups

[19] Mirza I, Jenkins R. Risk factors, prevalence, and treatment of anxiety and depressive disorders in Pakistan: systematic review. BMJ. 2004;328(7443):794. doi: 10.1136/bmj.328.7443.794 15070634 PMC383372

[20] Sindhu KV, Chandrashekarappa SM, Thambad M, Boralingiah P, Gopi A, Murthy MRN. Anxiety and depression among elderly tribal population of H.D. Kote, Mysuru, India. Archives of Mental Health. 2022;23(1):40–6. doi: 10.4103/amh.amh_103_21

[21] World Health Organization. Pakistan: Member State Profile. Mental Health Atlas 2020 2020 [cited 2025 Apr 24]. Available from: https://cdn.who.int/media/docs/default-source/mental-health/mental-health-atlas-2020-country-profiles/pak.pdf?sfvrsn=62378896_6&download=true

[22] Park LT, Zarate CA Jr. Depression in the Primary Care Setting. N Engl J Med. 2019;380(6):559–68. doi: 10.1056/NEJMcp1712493 30726688 PMC6727965

[23] Karami N, Kazeminia M, Karami A, Salimi Y, Ziapour A, Janjani P. Global prevalence of depression, anxiety, and stress in cardiac patients: A systematic review and meta-analysis. J Affect Disord. 2023;324:175–89. doi: 10.1016/j.jad.2022.12.055 36584710

[24] Bener A. High Prevalence of Depression, Anxiety and Stress Symptoms Among Diabetes Mellitus Patients. TOPJ. 2011;5(1):5–12. doi: 10.2174/1874354401105010005

[25] Somaili M, Kilani W, Sultan M, Kariri R, Majrashi E, Alfaifi N, et al. Prevalence of anxiety and depression among hypertensive patients: a systematic review. IJMDC. 2022;:178–85. doi: 10.24911/ijmdc.51-1631971689

[26] Gong L, Ma T, He L, Lin G, Zhang G, Cheng X, et al. Association between single and multiple cardiometabolic diseases and depression: A cross-sectional study of 391,083 participants from the UK biobank. Front Public Health. 2022;10:904876. doi: 10.3389/fpubh.2022.904876 35991068 PMC9386503

[27] Huang Z-T, Luo Y, Han L, Wang K, Yao S-S, Su H-X, et al. Patterns of cardiometabolic multimorbidity and the risk of depressive symptoms in a longitudinal cohort of middle-aged and older Chinese. J Affect Disord. 2022;301:1–7. doi: 10.1016/j.jad.2022.01.030 34999125

[28] Zhou Y, Kivimäki M, Lim CCW, Carrillo-Larco RM, Qi S, Wu X, et al. Bidirectional Associations Between Cardiometabolic Multimorbidity and Depression and Mediation of Lifestyles: A Multicohort Study. JACC Asia. 2024;4(9):657–71. doi: 10.1016/j.jacasi.2024.06.004 39371624 PMC11450941

[29] Sharif H, Sheikh SS, Thompson AM, Hashim M, Seemi T, Zaidi K, et al. Prevalence of Mental Disorders Among Patients with Multimorbidity Visiting Primary Care Settings in Slums of Karachi, Pakistan. J Prim Care Community Health. 2024;15:21501319241258658. doi: 10.1177/21501319241258658 38813988 PMC11143854

[30] Farooq S, Khan T, Zaheer S, Shafique K. Prevalence of anxiety and depressive symptoms and their association with multimorbidity and demographic factors: a community-based, cross-sectional survey in Karachi, Pakistan. BMJ Open. 2019;9(11):e029315. doi: 10.1136/bmjopen-2019-029315 31748286 PMC6887067

[31] Felez-Nobrega M, Haro JM, Koyanagi A. Multimorbidity, depression with anxiety symptoms, and decrements in health in 47 low- and middle-income countries. J Affect Disord. 2022;317:176–84. doi: 10.1016/j.jad.2022.08.110 36055525

[32] World Health Organization. Hypertension Pakistan 2023 country profile 2023 [cited 2025 April 27]. Available from: https://www.who.int/publications/m/item/hypertension-pak-2023-country-profile

[33] Fatmi Z, Kondal D, Shivashankar R, Iqbal R, Khan AA, Mohan D, et al. Prevalence of dyslipidaemia and factors associated with dyslipidaemia among South Asian adults: The Center for Cardiometabolic Risk Reduction in South Asia Cohort Study. Natl Med J India. 2020;33(3):137–45. doi: 10.4103/0970-258X.314005 33904416

[34] Samad Z, Hanif B. Cardiovascular Diseases in Pakistan: Imagining a Postpandemic, Postconflict Future. Circulation. 2023;147(17):1261–3. doi: 10.1161/CIRCULATIONAHA.122.059122 37093966 PMC10629490

[35] Sewpaul R, Mbewu AD, Fagbamigbe AF, Kandala N-B, Reddy SP. Prevalence of multimorbidity of cardiometabolic conditions and associated risk factors in a population-based sample of South Africans: A cross-sectional study. Public Health Pract (Oxf). 2021;2:100193. doi: 10.1016/j.puhip.2021.100193 36101622 PMC9461539

[36] NovoPsych. Depression Anxiety Stress Scales – Short Form (DASS-21) [cited 2024 September 22]. Available from: https://novopsych.com.au/assessments/depression/depression-anxiety-stress-scales-short-form-dass-21/

[37] Lovibond SH, Lovibond PF. Depression Anxiety Stress Scales (DASS--21, DASS--42): APA PsycTests; 1995 [cited 2025 Apr 24]. Available from: https://psycnet.apa.org/doiLanding?doi=10.1037%2Ft01004-000

[38] Waqar H, Amir G. Translation, adaptation and validation of Depression, Anxiety and Stress Scale in Urdu. Insights Depress Anxiety. 2020;4(1):001–4. doi: 10.29328/journal.ida.1001011

[39] Aslam N, Kamal A. Translation, validation and effectiveness of depression, anxiety and stress (DASS-21) in assessing the psychological distress among flood affected individuals. J Pak Psychiatr Soc. 2019;14(4):16–20.

[40] Khan UI, Shah S, Qureshi A, Viswanathan S, Merchant AT, Virani SS, et al. Burden of cardiometabolic diseases and depression in a low-income, urban community in Pakistan: a cross-sectional survey. BMC Public Health. 2025;25(1):757. doi: 10.1186/s12889-025-21939-6 39994704 PMC11852865

[41] Dean AG, Sullivan KM, Soe MM. OpenEpi: Open Source Epidemiologic Statistics for Public Health 2013 [cited 2023 Sep 1]. Available from: https://www.openepi.com/Menu/OE_Menu.htm

[42] DASS-21 [cited 2025 Apr 29]. Available from: https://maic.qld.gov.au/wp-content/uploads/2016/07/DASS-21.pdf

[43] Kivimäki M, Kuosma E, Ferrie JE, Luukkonen R, Nyberg ST, Alfredsson L, et al. Overweight, obesity, and risk of cardiometabolic multimorbidity: pooled analysis of individual-level data for 120 813 adults from 16 cohort studies from the USA and Europe. Lancet Public Health. 2017;2(6):e277–85. doi: 10.1016/S2468-2667(17)30074-9 28626830 PMC5463032

[44] Basit A, Younus BB, Waris N, Fawwad A, members NDSP. Prevalence of tobacco use in urban and rural areas of Pakistan; a sub-study from second National Diabetes Survey of Pakistan (NDSP) 2016 - 2017. Pak J Med Sci. 2020;36(4):808–15. doi: 10.12669/pjms.36.4.1705 32494279 PMC7260914

[45] Yin J, Ma T, Li J, Zhang G, Cheng X, Bai Y. Association of mood disorder with cardiometabolic multimorbidity trajectory and life expectancy, a prospective cohort study. J Affect Disord. 2022;312:1–8. doi: 10.1016/j.jad.2022.06.003 35690125

[46] Otieno P, Asiki G, Wilunda C, Wami W, Agyemang C. Cardiometabolic multimorbidity and associated patterns of healthcare utilization and quality of life: Results from the Study on Global AGEing and Adult Health (SAGE) Wave 2 in Ghana. PLOS Glob Public Health. 2023;3(8):e0002215. doi: 10.1371/journal.pgph.0002215 37585386 PMC10431646

[47] UN Women. National Report on the Status of Women in Pakistan, 2023 - A Summary: UN Women; 2023 [cited 2024 22 Sep]. Available from: https://pakistan.unwomen.org/sites/default/files/2023-07/summary_-nrsw-inl_final.pdf

[48] Jamil S. From digital divide to digital inclusion: Challenges for wide-ranging digitalization in Pakistan. Telecommunications Policy. 2021;45(8):102206. doi: 10.1016/j.telpol.2021.102206

[49] Rahim S, Qutoshi SB, Abida S, Muhammad F, Hussain I. Access and Use of Mobile Phone in Daily Life Activities by Rural Women of Gilgit-Baltistan, Pakistan. Mobile Information Systems. 2020;2020:1–11. doi: 10.1155/2020/8835877

[50] GSMA. The Mobile Gender Gap Report 2020 [cited 2024 23 Sep]. Available from: https://www.gsma.com/r/gender-gap-2020/

[51] Statista. Percentage of individuals worldwide reporting moderate to severe symptoms of stress, depression, and anxiety in 2022, by age group: Statista; 2022 [cited 2024 24 Sep]. Available from: https://www.statista.com/statistics/1400861/percentage-of-individuals-reporting-symptoms-of-stress-depression-anxiety-by-age-group-worldwide/

[52] Lorant V, Deliège D, Eaton W, Robert A, Philippot P, Ansseau M. Socioeconomic inequalities in depression: a meta-analysis. Am J Epidemiol. 2003;157(2):98–112. doi: 10.1093/aje/kwf182 12522017

[53] GBD 2019 Mental Disorders Collaborators. Global, regional, and national burden of 12 mental disorders in 204 countries and territories, 1990-2019: a systematic analysis for the Global Burden of Disease Study 2019. Lancet Psychiatry. 2022;9(2):137–50. doi: 10.1016/S2215-0366(21)00395-3 35026139 PMC8776563

